# ‘So, this feels very different and, you know, kindly’: a mixed methods study of arts on prescription in secondary care

**DOI:** 10.1093/eurpub/ckag151

**Published:** 2026-08-17

**Authors:** Nicola J Holt, Donna Baber, Caroline Elliott

**Affiliations:** Department of Social Sciences, University of the West of England (UWE), Frenchay Campus, Bristol, United Kingdom; Fresh Arts, North Bristol NHS Trust (NBT), Southmead Hospital, Westbury-on-Trym, Bristol, United Kingdom; Department of Social Sciences, University of the West of England (UWE), Frenchay Campus, Bristol, United Kingdom

## Abstract

Arts on Prescription (AoP) programmes occur within primary care and community settings. The aim of this study was to examine the impact of AoP delivered in secondary care, on patient wellbeing, social connection, and experiences of care. A mixed-methods design was used. Quantitative data were collected from 61 patients referred to AoP through secondary care outpatient services for cancer, chronic pain, weight management, breathlessness, or neuromuscular conditions. Wellbeing and loneliness were assessed pre- and post-participation in the AoP programme. Mood and loneliness were assessed before and after each arts workshop. Qualitative methods focused on experiences of AoP and were collected through semi-structured interviews and focus groups (*n *= 12) and analysed using thematic analysis. Wellbeing scores increased significantly over the course of the programme, alongside reductions in loneliness. Arts workshops increased relaxation, contentment, and social connection. Thematic analysis led to four themes: (i) *Connection to others—*feeling supported, inspired, and understood; (ii) *Making space—*providing relaxing, protected time; (iii) *Becoming—*renewed confidence, and opening to new opportunities; and (iv) *Relationships with healthcare—*reframing interactions with secondary care, validating referrals, and supporting the management of health conditions. AoP in secondary care demonstrates potential as an effective approach, replicating effects in primary care, including improved wellbeing and social connection. However, additional merits in this context relate to healthcare relationships, and using the arts to manage health conditions. Embedding AoP within secondary care may reduce burden on services by supporting psychosocial needs alongside medical care.

## Introduction

Arts on prescription (AoP) is a form of social prescribing, involving referrals to arts programmes (rather than to other activities such as sport) [1]. Social prescribing (SP) occurs when trusted professionals (e.g. doctors, social workers) signpost people to community resources and groups with the hope that engagement will improve their psychosocial wellbeing [2]. SP is offered for various reasons: psychosocial (e.g. social isolation); mental health (e.g. low to moderate levels of stress, anxiety or depression); and physical health (e.g. chronic pain) [3]. It recognizes the social determinants of health, for instance, the role of social isolation in stress, anxiety, and depression [4, 5]. In addition to improving health outcomes, SP is expected to reduce demand on NHS services by decreasing patient contact with healthcare providers [6]. SP usually occurs in community settings and primary care, however, its use is being expanded in scope, for example, being piloted in schools and universities [7]. This article examines the use of AoP within secondary care (specialized care, usually in a hospital, following a referral from primary care) [8].

Previous research on AoP in primary care suggests significant improvements in psychosocial wellbeing, and reduced symptoms of anxiety and depression [9–11]. A recent meta-analysis of AoP outcomes shows an overall significant increase in wellbeing [1]. Qualitative studies suggest that AoP is seen as meaningful, supporting social connection, improved confidence and wellbeing, and providing progression opportunities [12–14]. While there are limitations with the evidence base (e.g. a reliance on observational data and a lack of longitudinal work), existing research consistently supports the efficacy of AoP as meaningful and efficacious.

AoP could be useful in the context of secondary care. Consideration of this is especially pressing given high levels of social isolation, anxiety, and stress amongst patients in secondary care with chronic health conditions [15, 16]. While there is limited research using AoP in secondary care, AoP has been reported to improve the wellbeing and mental health of people experiencing chronic pain and cancer in evaluation reports [17, 18].

This project aims to contribute to this evidence base using a mixed-methods approach to examine the impact of AoP in secondary care among patients receiving treatment for cancer, chronic pain, chronic breathlessness, neuromuscular conditions, and weight management. Based on previous AoP research in primary care, it was hypothesized that patient wellbeing would increase from pre- to post-participation in the AoP programme [9]. Further, it was hypothesized that participants would report reduced loneliness and improved mood following each arts workshop [10, 19]. Qualitative interviews and focus groups were used to explore participants’ experiences of AoP in secondary care and to provide insight into the unique impact of the programme within this setting.

## Methods

### Participants

Quantitative data were collected from 61 individuals (54 female, 6 male, 1 unspecified), aged 26–82 years (M = 55.39, SD = 12.34). Most participants identified as White-British (*n* = 47), with others identifying as Mixed (*n* = 2), Black (*n* = 1), White-Irish (*n* = 2), White-Eastern European (*n* = 1), Other (*n* = 1), White-Other (*n* = 2), and five participants preferring not to disclose. Participants were referred to the following groups: Cancer (*n* = 17), Weight Management (*n* = 9), Chronic Pain (*n* = 16), Chronic Breathlessness (*n* = 10), and Neuromuscular Conditions (*n* = 9). Qualitative data were provided by 12 individuals (all female, aged 26–65 years), including 3 interviews (1 Cancer; 2 Chronic Pain) and 9 participants in focus groups (5 Neuromuscular Conditions; 4 Weight Management).

### Design

A mixed-methods design was used. The quantitative component utilized an observational pre-post-design, with repeated measurements across two time series: pre- to post-AoP programme (wellbeing and loneliness); and pre- and post-arts workshops (mood and state loneliness). The qualitative component consisted of semi-structured interviews and focus groups, analysed with reflective thematic analysis.

### Materials

#### Person-level measures

*Warwick Edinburgh Mental Wellbeing Scale, WEMWBS*: A 14-item scale measuring psychological wellbeing, including connection to others, self-esteem, positive affect and clarity of cognition [20]. The scale has shown excellent internal consistency, construct validity, and responsiveness to change [21, 22]. A score of 40 or below has been interpreted as indicative of probable depression, and 44 or below of possible depression [23, 24].

*Direct Measure of Loneliness, DMoL*: A single item measure of loneliness (“How often do you feel lonely?”) with a 5-point response scale, where a score of 5 indicates extreme loneliness [25]. The DMoL demonstrates convergent validity with established multi-item loneliness scales [19, 25].

*Campaign to End Loneliness Measurement Tool, CtELMT*: Co-designed with service users, this scale uses sensitive language to reduce distress [26]. Its 3-item scale measures satisfaction with social connection and relationships. It has demonstrated adequate convergent validity [19]. Possible scores range from 0 to 12, where scores of 10–12 are thought to indicate intense loneliness.

#### State (experiential level) measures

*Short Mood Scale, SMS*: A 6-item scale measuring three dimensions of mood—contentment (vs. sadness), calmness (vs. anxiety or stress), and feeling awake and alert (vs. a lack of energy) [27]. It has shown reliability and sensitivity to individual change [10, 18, 28].

*State loneliness:* A single item: ‘How lonely do you feel at the moment?’ [29]. It has shown adequate validity and reliability [18].

### Procedure

Participants were referred from clinics to participate in AoP at Southmead Hospital, data being collected from January 2021 to February 2023. AoP was delivered by Fresh Arts, the arts programme for Southmead Hospital, North Bristol NHS Trust. Through creative writing, print making, visual art, and poetry, the programme aims to support patients with chronic long-term illness to better self-manage their condition and promote self-care by decreasing experiences of stress, anxiety, and social isolation. AoP consisted of weekly 2-hour-long arts workshops for a 6-week-long period. For cancer patients the workshops focused on creative writing (poetry and prose). Other groups participated in visual art activities (painting, drawing, felting, quilling, and collage). All workshops were led by trained arts for health facilitators, and were designed to enable creative play without judgement, provide enjoyment, and social connection. The weight management and neuromuscular conditions groups were delivered online to reduce barriers to participation, including those related to mobility, travel, and health, while offering the same visual art activities.

All participants on the programmes were invited by arts for health facilitators to read a participant information sheet about the research, and, if interested in taking part, provide informed consent. Participants were invited to complete person-level measures at the start and end of the 6-week-long programme, and state measures at the start and end of each arts workshop. All data were contributed anonymously, being cross-referenced with a participant generated unique code (created in response to prompts).

Participants were invited to take part in qualitative research at the end of the 6-week-long programmes, providing informed consent, including for their data to be recorded, transcribed, and used for analysis and dissemination. Three focus groups were facilitated by D.B. and C.E., in the art space at Southmead Hospital, each including two to four participants. Interviews were conducted one-to-one, through Teams or Zoom, with N.J.H. Participants from all groups could choose their preferred option for participation. Both methods used a semi-structured schedule focused on experiences and perceived impact of AoP, and encouraged the sharing of artwork made during the programme to facilitate discussion. Focus groups and interviews ranged from 45-65 minutes in duration. Audio data were recorded and transcribed by C.E., N.J.H., and L.G., and were coded and analysed by C.E. and N.J.H. using inductive reflexive thematic analysis [30, 31]. This flexible method was used to identify and interpret patterns of shared meaning within the data. Analysis was conducted at the semantic level from a contextualist perspective, with ongoing discussions used to support reflexive engagement with the data and theme development.

Ethical approval was granted by the University of the West of England’s Ethics Committee (reference number: HAS.17.07.197).

## Results

### Quantitative results

Because observations were nested within participants, multilevel modelling was used to account for the non-independence of observations and missing data [32]. For the programme-level analyses, 120 observations of wellbeing and loneliness (Level 1) were nested within 61 participants (Level 2). For the workshop-level analyses, 479 observations of mood (hedonic tone, tense arousal, and energetic arousal) and state loneliness (Level 1) were nested within the same 61 participants (Level 2).

#### Wellbeing and loneliness scores from pre- to post-art programmes

On average wellbeing increased from 41.16 to 47.82, an average increase of 6.67 units (where a minimum increase of 3 units is thought to indicate meaningful change) [21]. All subgroups showed an increase in wellbeing across the AoP programmes (ranging from 3 to 9 units). A multi-level analysis was conducted (with a random intercept), predicting wellbeing with time (pre- and post-programme) (as a fixed factor). The wellbeing increase over time was statistically significant (as detailed in Table 1). Mean levels of loneliness (CtELMT; DMoL) decreased significantly across the programme (as detailed in Table 1).

**Table 1:** Programme-level and workshop-level outcomes for AoP participants, showing changes in outcomes over time.

Outcome measure	Pre-mean (SD)	Post-mean (SD)	β	SE	*t* (df)	*P*	Cohen’s *d*
Programme-level outcomes							
Wellbeing (WEMWBS)	41.16 (10.53)	47.82 (9.74)	3.34	0.53	6.29 (51.16)	<.001	1.76
Loneliness (CtELMT)	4.97 (2.71)	4.23 (2.56)	−0.45	0.18	−1.44 (56.65)	.018	−0.38
Loneliness (DMoL)	3.66 (1.08)	3.25 (1.07)	−0.19	0.06	−3.15 (51.83)	.003	−0.87
Workshop-level outcomes							
Relaxation (SMS)	96.99 (41.73)	145.13 (35.07)	23.92	1.57	15.22 (422.19)	<.001	1.48
Contentment (SMS)	104.27 (38.41)	136.62 (34.74)	16.15	1.30	12.41 (416.33)	<.001	1.22
Energy (SMS)	81.35 (42.36)	95.67 (46.89)	6.80	1.69	4.03 (421.81)	<.001	0.39
State loneliness	35.27 (24.91)	19.69 (19.27)	–7.23	0.69	–10.50 (398.81)	<.001	−1.05

SD = standard deviation. Time refers to pre-post programme for programme level outcomes and pre-post workshops for workshop level outcomes. For state measures scores range from 0 to 200 (for relaxation, contentment and energy) and from 0 to 100 for loneliness.

#### State changes during arts workshops

Multi-level analyses were conducted (with a random intercept), with state variables as dependent variables and time (pre- and post-workshops) as a fixed factor, to test whether participants reported improved mood and reduced loneliness during the arts workshops. There were statistically significant improvements in all dimensions of mood (as detailed in Table 1), with increased relaxation, contentment, and energy. Participants also felt significantly less lonely after the workshops.

### Qualitative results

Four themes were identified through the stages of thematic analysis, detailed in Table 2: (i) ‘Connection to others’, where participants felt supported by others; (ii) ‘Making space’, articulating the benefits felt from the structure of the art programme that gave permission to relax and focus on art making; (iii) ‘becoming’, which described experiences of change across the AoP programme, (becoming more confident, less self-critical, and developing positive identities); and (iv) ‘relationships with healthcare’, which had two subthemes: ‘the hospital as a trusted space’ and ‘art as a health behaviour’.

**Table 2:** Themes, example codes, and quotes from interviews and focus groups.

Themes and subthemes	Example codes	Example quotes
Connection to others	Feeling a part of something In the same boat Inspiration from others Relaxing with others Sharing Pride in artwork	‘So, we’re all coming from the same sort of situation, obviously everybody is different, you know, but I think there’s something [sigh] reassuring to know that other people have the same battles’ ‘But by seeing what other people were doing and sharing ideas and so on found, that’s really quite inspiring and that really helped me’.
Making space	Protected time Relaxation Concentration A happy place Forgetting	‘Erm so yeah, it’s that yeah that sort of mental space. And I really liked that someone said happy place, yeah [laughs] saying that here is my happy place’. ‘it has helped far more than any of the other things that I have been doing [laughs]. N…in the way that it actually has taken my mind off stuff and helped me to focus on something else, and um, and wh, wh, wh, is what I needed’.
Becoming	Change of attitude More confident Being an artist Springboard to something new	‘It’s encouraged me to try new hobbies, erm, to meet other people and um, to not be too critical of myself or how well I’m doing’. ‘So yes, it’s, it’s helped me to to feel more confident in in that respect and not to rubbish myself all the time’.
Relationships with healthcare The hospital as a trusted space Art as a health behaviour	Different type of treatment Kindly hospital Trusting hospital referrals Managing health conditions Self-care Self-regulation	‘I just remember it being absolutely terrifying [hospital], and, and just hating it so much, you know. So, so, this feels like very different and like very, you know kindly’. ‘…in these struggles of living with a kind of condition has, which is a physically invisible condition which you do not see[chuckles] you know? Erm, erm and I think that this has enabled me to actually go “what do I need and what do I need to nurture myself?”’

#### Connection to others

This theme focused on the benefits participants reported from spending time with others in the group and feeling a part of something larger than themselves. The group context provided a safe space for expression and collaboration, which participants described as motivating and enjoyable. Further, working with people with similar health conditions was described as reassuring, as participants felt understood by others and ‘in the same boat’. This emphasized the comfort gained from working with others with a similar diagnosis and experience.

*‘There is also just an understanding, although we never spoke about it, that we were there because we had chronic conditions, because we had things in our life that weren’t easy. You don’t want to talk about it, it’s your escape from all that, but in the group you feel that you are understood’.*

Participants described finding inspiration from each other, sharing ideas, and enjoying seeing the progress made by others. This not only fostered interest and excitement in art making, but helped to develop a sense of pride, and recognition of their achievements on the programme.

#### Making space

Participants discussed how the weekly art group provided a helpful structure, protecting time and giving permission to use that time to make art. In this space, participants felt able to relax, in a way that they had previously found difficult. The 2 hours of art making, each week, was described as ‘just for me’, a space that helped people ‘escape’ from worries and difficulties.

*‘So yeah this, this sort of brought things a bit of calmness, even if it was for just these couple of hours, and, but it also. I don’t know it just sort of like, it it was almost like a de-stress of work, it all sort of left my body and I could go, … I can sort of feel myself going up, and then there’s a [exhales] calm down, calm, down. But yeah it’s um it’s definitely made me a bit more calm and about more relaxed’.*

This space was also described as ‘healthy’ and ‘happy’, and could extend into everyday life. The workshops helped to create a ‘mental space’ that participants learnt to access in their own time, helping to give respite from anxiety, rumination, and stress, through absorption in art-making.

#### Becoming

Participants discussed how their sense of self, behaviours, or identities changed over the course of the programme, taking them on a ‘journey of becoming’. This included being more adventurous, being open to trying new things and taking opportunities in life, being less hindered by self-criticism or anxiety and having a new sense of confidence.

*‘You know, I just go and try my best, you know, do what makes me happy. What works for me and not be like “Oh I can’t do that”. “I’m not good at art, sports, etcetera”… It’s almost like an attitude change’.*

Participants also discussed thinking of themselves as an artist because of attending AoP, which gave a sense of pride and a new, positive, sense of identity.

*‘… I had a piece exhibited at the art gallery in a little exhibition…which was lovely so there was like, it was lovely to go and instead, of like “Well how did you hear about it?” It was like, “well I’m one of the artists!” [laughs] a kind of defining moment [all laugh]. I call myself an artist’.*

Participants noted a shift in how they viewed themselves, both in terms of artistic identity and broader personal growth, which they attributed to experiences gained during the AoP programme.

#### Relationships with healthcare

*The hospital as a trusted space*: Participants discussed how AoP changed their relationships with the NHS or hospitals, and how their experience of AoP contrasted with previous experiences in medical settings. They described feeling supported by the programme, perceiving kindness, that made them feel more connected ‘to the hospital’.

*‘…sort of going to the hospital’s always sort of bit scary bit, horrible. I don’t really like it. And, and then this made it seem much more personally accessible. So actually, I think it’s sort of like has changed my relationship with the hospital. Changed how I see my relationship with the department’.*

Being referred to AoP by and within the hospital was important to participants. They discussed how having a referral validated attending the art classes, to themselves and to others. Because the referral came from a medical professional, attending the arts workshops was given more importance.

*‘…because this was offered as a… like a bit of medical information. I could sort of actually ask my manager, could I take time out to go. And um, which she agreed to because, um I wasn’t being very functional’.*

*Art as a health behaviour*: AoP was described as helping with health conditions in various ways, such as managing anxiety, improving hand mobility, and managing chronic pain and eating behaviours (by using art-making as a tool to regulate emotions and attention). Participants also described how they felt able to give more time to ‘themselves’ and doing more activities that promoted health and wellbeing.

*‘I find that it’s helped me in that, I will sit at home on an evening and watch TV programmes and I will eat because I’m just sat there like that [imitates eating] I’m like… when I’m not thinking about it and now, I don’t eat I do crafts’*

*‘It’s not easy, living with a long-term condition, particularly with an unseen one, and people don’t get it. But when I do the art it kind of melts away. I can forget the pain. I can forget how tired I am. I just I can escape, escape from my own thoughts a little bit. It gives me a space that’s good for me and I feel invigorated by it. So, that’s my “go to” now’.*

Engagement in AoP influenced participants’ perceptions of healthcare and self-management of conditions, with skill and wellbeing gains being translated into more balanced routines and healthier behaviours.

## Discussion

This study extends the literature on AoP by using a mixed-methods design to examine its impact in secondary care. Findings suggest that AoP in secondary care is associated with improvements in wellbeing and social connection like those reported in the primary care and community settings, while also highlighting its potential to positively influence patients’ relationships with healthcare services.

Outcomes suggest that AoP can improve wellbeing and reduce loneliness among people receiving secondary care for a range of conditions (cancer, chronic pain, breathlessness, weight management difficulties, and neuromuscular conditions). Participants reported improvements in wellbeing and reductions in loneliness, supporting the argument that AoP has measurable psychosocial benefits [1, 9, 10, 18]. In this study, average wellbeing increased from a level indicative of possible depression to above this threshold at the end of programmes. This magnitude of change is consistent with that reported in the wider literature on AoP in primary care [1, 23, 24].

Participants were significantly more relaxed, content, energetic, and felt less lonely, immediately following the arts workshops. This aligns with research examining the psychosocial mechanisms of AoP, through shifts in mood, attention, and belonging [10, 18, 33]. These immediate benefits might feed into longer-term improvements in wellbeing [10].

Crucially, the qualitative outcomes triangulated with the quantitative outcomes. The three themes: ‘Connection to others’, ‘Making space’, and ‘Becoming’ illustrate how AoP programmes were experienced as transformative and positive for participants with various health conditions. AoP provided social support and bonding, and engagement with art making helped participants to enter a state of relaxed absorption characterized by reduced stress and worry. Across the programme experiences of making and creativity, helped to foster confidence and led to new opportunities. These three themes mirror those from AoP in primary care [1, 11, 12]. However, the importance of sharing space with others who had similar health conditions was emphasized in the present study, fostering mutual understanding based on first-hand experience. This may differ from many AoP groups in primary care, where participants often have more varied reasons for referral [9].

The most original finding from the thematic analysis was the theme, ‘relationships with healthcare’. The first sub-theme illustrated how art became a tool for managing health conditions in daily life, for example, by distracting from symptoms and reducing anxiety, while encouraging self-care and self-compassion. Consideration of how AoP aligns with approaches to the self-management of specific chronic conditions may help further develop the use of art as a health behaviour [34]. The second sub-theme illustrated how AoP affected participants’ relationship with secondary care itself. The experience of AoP led participants to reframe their perceptions of ‘the hospital’ as more positive and caring, potentially impacting future healthcare interactions and relationships. This aligns with work on the ‘humanizing’ impact of art in hospitals more widely, which may have positive health impacts [35, 36]. Whereas secondary care can be experienced as anxiety-provoking or impersonal, AoP was described as supportive, humanizing, and validating. The act of being ‘referred’ by clinicians further legitimized participation, allowing patients to prioritize engagement and, in some cases, negotiate adjustments at work. This suggests that AoP in secondary care may not only improve patient wellbeing but also strengthen trust in and engagement with healthcare services.

### Implications for practice and policy

The present study suggests that AoP can be embedded successfully within secondary care. In line with NHS priorities around personalized care and non-clinical interventions, secondary-care AoP programmes could offer valuable adjuncts to medical treatment, particularly for patients with chronic and complex conditions where psychosocial support is essential [37]. The group-based model offers potential efficiency gains by reducing isolation, enhancing self-management, and potentially alleviating demand on clinical services [38]. AoP in secondary care may help address inequalities in access to creative health interventions, particularly for those whose health status restricts community participation. However, there is limited evidence for SP in secondary care, and in relation to chronic health conditions, and further research is required to provide the evidence required for best practice and implementation [1, 39].

## Strengths and limitations

A key strength of this study was the integration of quantitative and qualitative methods, which offered rich insights into both outcomes and lived experiences. However, several limitations should be acknowledged. First, sample sizes were modest, and the cohort included multiple health conditions and deliveries (e.g. visual arts and creative writing, in-person and online delivery). Second, with observational designs causal conclusions remain tentative, since change over time could be attributed to other factors (such as seasonal changes). Studies incorporating control groups (including ‘n of 1’ designs) would be useful. Thirdly, the participants (especially in the qualitative component) had a limited demographic, being mostly female, over 50 years of age, and White. Finally, since involvement in the research was optional, participants represent a self-selecting group. This may introduce bias, for example, being more willing to participate in focus groups if they had had a positive experience of AoP.

Future research should aim to replicate these findings with larger samples across multiple sites, explore cost-effectiveness, and compare outcomes across different groups (and art forms), with outcome measures relevant to specific health conditions (e.g. pain self-efficacy). Longitudinal follow-up would also be valuable to determine whether wellbeing benefits are sustained over time.

## Conclusion

AoP within secondary care was associated with significant improvements in wellbeing and reductions in loneliness, alongside immediate mood and social benefits. Thematic analysis illuminated how AoP fostered social connection, created protected psychological space, supported identity change, encouraged health behaviours and positively reshaped patients’ perceptions of healthcare environments.

These findings highlight the potential of AoP as a valuable adjunct within secondary care, offering psychosocial support alongside clinical treatment for people living with chronic conditions. Embedding AoP within secondary care could play an important role in delivering more holistic, patient-centred care, while contributing to NHS goals of prevention, self-management, and improved patient experience [40].

## Data Availability

The data underlying this article cannot be shared publicly due to ethical issues, respecting the privacy of individuals that participated in the study. Key Points Arts on Prescription delivered through secondary care was associated with improved wellbeing and reduced loneliness among patients with chronic health conditions. Arts workshops produced immediate improvements in mood and social connection. Participants described Arts on Prescription in secondary care as improving their relationship with healthcare services and supporting new health behaviours Arts on Prescription in secondary care, for patients with long-term or complex conditions, can reduce isolation and distress. Scaling Arts on Prescription programmes within secondary care has potential public health benefits by enhancing patient experience and supporting wellbeing, with future research needed to examine effects on service use.
